# Differences Between Emergency Medicine Residency Programs That Accept the Comprehensive Osteopathic Medical Licensing Examination of the United States and Those That Prefer or Only Accept the United States Medical Licensing Examination

**DOI:** 10.7759/cureus.22704

**Published:** 2022-02-28

**Authors:** Dhimitri A Nikolla, Breanne M Jaqua, Tanner Tuggle, Zachary J Jarou

**Affiliations:** 1 Department of Emergency Medicine, Saint Vincent Hospital, Allegheny Health Network, Erie, USA; 2 Department of Clinical Education, School of Osteopathic Medicine in Arizona/Andrew Taylor Still University, Mesa, USA; 3 Department of Emergency Medicine, St. Joseph Mercy Ann Arbor Hospital, University of Michigan Medical School, Ann Arbor, USA

**Keywords:** postgraduate residents, osteopathic medical student, emergency medicine residency, postgraduate education, emergency medicine resident

## Abstract

Introduction

The Comprehensive Osteopathic Medical Licensing Examination of the United States (COMLEX-USA) is a three-part series of examinations similar to the United States Medical Licensing Examination (USMLE) that osteopathic medical students must pass for medical licensure. Osteopathic students are not required to take the USMLE, but during the residency admission process, many emergency medicine (EM) residency programs will not consider osteopathic applicants who only take the COMLEX-USA. Therefore, we examined program-level characteristics between programs that accept the COMLEX-USA alone for osteopathic applicants and those that prefer the USMLE using free, publicly available online databases and residency program websites.

Methods

Emergency Medicine Residents’ Association (EMRA) Match was the primary database used; however, missing exam preferences were supplemented from Fellowship and Residency Electronic Interactive Database Access (FREIDA) and individual program websites. Program characteristics were compared between EM residencies that accept the COMLEX-USA and those that prefer the USMLE using the Chi-square test for categorical variables and the Mann-Whitney test for interval variables.

Results

Two hundred sixty of the 278 EM programs in the dataset were included in the analyses. One hundred and seven programs reported preferring the USMLE, while 151 reported accepting the COMLEX-USA alone. Programs differed by the educational environment of the primary training site (p <0.001), number of Standardized Letter(s) of Evaluation (SLOE) needed for an interview (p = 0.042), emergency department (ED) shift length (p = 0.021), former American Osteopathic Association accreditation (p <0.001), percentage of osteopathic residents (p <0.001), annual ED volume (p = 0.001), number of intern positions (p <0.001), and number of elective weeks (p = 0.028).

Conclusion

EM residency programs that reported accepting the COMLEX-USA alone for osteopathic applicants differed from those that prefer the USMLE. Therefore, osteopathic medical students interested in EM should consider these differences when deciding whether to take the USMLE.

## Introduction

The training pathway for students enrolled at osteopathic medical schools to match into residency and obtain a license to practice medicine independently is similar to that of students enrolled at allopathic medical schools, with the exception that osteopathic trainees are required to pass all levels of the three-part Comprehensive Osteopathic Medical Licensing Examination of the United States (COMLEX-USA) [1]. The COMLEX-USA is similar to the three-part United States Medical Licensing Examination (USMLE) that allopathic medical students are required to complete for medical licensure; however, the COMLEX-USA contains osteopathic content that the USMLE does not [2]. The USMLE is not a requirement for osteopathic trainees and taking it was unnecessary for those applying to American Osteopathic Association (AOA) accredited residency programs before the Single Accreditation System. However, the Single Accreditation System transitioned all AOA programs to Accreditation Council for Graduate Medical Education (ACGME) accreditation, placing formerly osteopathic and allopathic residency training programs under the same accrediting body [3]. Before the transition, osteopathic applicants who did not take the USMLE and applied to allopathic emergency medicine (EM) programs were less likely to match [3-6]. Although scores between the two exams are strongly correlated, they are not interchangeable, and EM residency programs frequently use USMLE scores to screen applicants for residency interviews [7-10]. The American Medical Association (AMA) promotes equal acceptance of both exams by United States (US) residency programs [11]. However, the Council of Emergency Medicine Residency Directors (CORD) still recommends that osteopathic applicants take the USMLE as it may improve the applicant’s chances of matching into EM by allowing a more direct comparison between them and their allopathic peers [6].

Nevertheless, the USMLE is a high-stakes series of exams with a significant time and financial commitment to the osteopathic applicant. It often requires additional study as the USMLE and COMLEX-USA are not identical, and some applicants may be at risk of failing one or both by diverting time and effort from focusing on just the COMLEX-USA. Therefore, only 60.7% of osteopathic seniors in the 2020 match reported taking the USMLE Step 1 [12].

While the proportion of osteopathic applicants who recently matched into EM without the USMLE was about 75% [12], not completing the USMLE may have significant limitations on the types of programs or locations available to them. It is unknown whether programs, after the Single Accreditation System transition, differ significantly between those which accept the COMLEX-USA alone for osteopathic applicants and those that prefer the USMLE. The objective of this study was to describe and compare the characteristics of EM residency training programs based upon their exam preference to help inform osteopathic applicants’ decisions to take the USMLE. An abstract with limited results was presented as an on-demand virtual presentation at the 2021 American College of Emergency Physicians Research Forum from October 25-28, 2021.

## Materials and methods

Study design

Emergency Medicine Residents’ Association (EMRA) Match is an interactive, collaborative, and filterable catalog that allows medical students applying to EM residencies to search for programs using various parameters, including whether a residency program accepts the COMLEX-USA alone for osteopathic applicants or prefers the USMLE [13,14]. Residency programs provide the information for their respective programs, and the online tool is free and publicly available. We performed a retrospective, cross-sectional analysis of data from EMRA Match. However, exam preference was missing for a substantial proportion of programs; therefore, missing exam preferences were added to the study dataset if available on AMA’s Fellowship and Residency Electronic Interactive Database Access (FREIDA), a publicly available online database of residency programs similar to EMRA Match, or from the individual residency program’s website [15]. Findings of this study are reported in accordance with the Strengthening the Reporting of Observational Studies in Epidemiology (STROBE) Guidelines [16].

Data collection

The EMRA Match data was downloaded on August 9, 2021. The dataset was augmented to include whether a program has had prior accreditation by the AOA [17]. US Census Bureau Region was added for the primary training site of each program [18]. Missing exam preferences were added to the study dataset if available on FREIDA or from the program website if not available on FREIDA. Programs were classified as preferring USMLE if they reported preferring or only accepting USMLE scores in EMRA Match, reported not requiring COMLEX-USA scores for osteopathic applicants, or explicitly requiring only USMLE scores in FREIDA, or only requested USMLE scores or explicitly required only USMLE scores on their program website. Programs were classified as accepting COMLEX-USA if they reported accepting USMLE or COMLEX-USA scores in EMRA Match, reported requiring COMLEX-USA scores for osteopathic applicants in FREIDA, or requested COMLEX-USA for osteopathic applicants on their program website. Programs with missing exam preferences from all three sources were noted as missing.

Study population

We studied factors unique to US EM residency training programs from pre-existing, publicly available online databases and individual program websites [14,15].

Outcome measures

The two cohorts of EM residency programs, those that accept COMLEX-USA alone for osteopathic applicants and those that prefer the USMLE, were compared across multiple variables. Variables included geographic region, the educational environment of the primary training site (i.e., community, county, or university), whether the program has a USMLE Step 1 minimum score, whether the program accepts applicants with USMLE Step 1 failures, whether the program allows moonlighting, the number of Standardized Letter(s) of Evaluation (SLOE) need for an interview, program length (i.e., three or four years), emergency department (ED) shift length, ED shifts per month, former AOA accreditation, the percentage of osteopathic residents, annual ED volume, percent of intern rotations that are off service, number of intern positions, number of elective weeks, and number of critical care weeks.

Data analysis

The Chi-square test was used to compare categorical variables. Comparisons between continuous variables were made using the Mann-Whitney test since all continuous variables were non-parametric as determined by the Shapiro test. The Chi-square test for trend in proportions was used to test for the significance of the trend. A p-value <0.05 was considered significant. Missing data for each program characteristic was reported for all variables and included as a factor for categorical variable comparisons. The EMRA Match dataset was downloaded as a Microsoft® Excel® (Microsoft Corporation, Redmond, WA) file, and analyses were performed with RStudio® (RStudio, PBC, Boston, MA, Version 1.4.1106).

## Results

**Figure 1:**
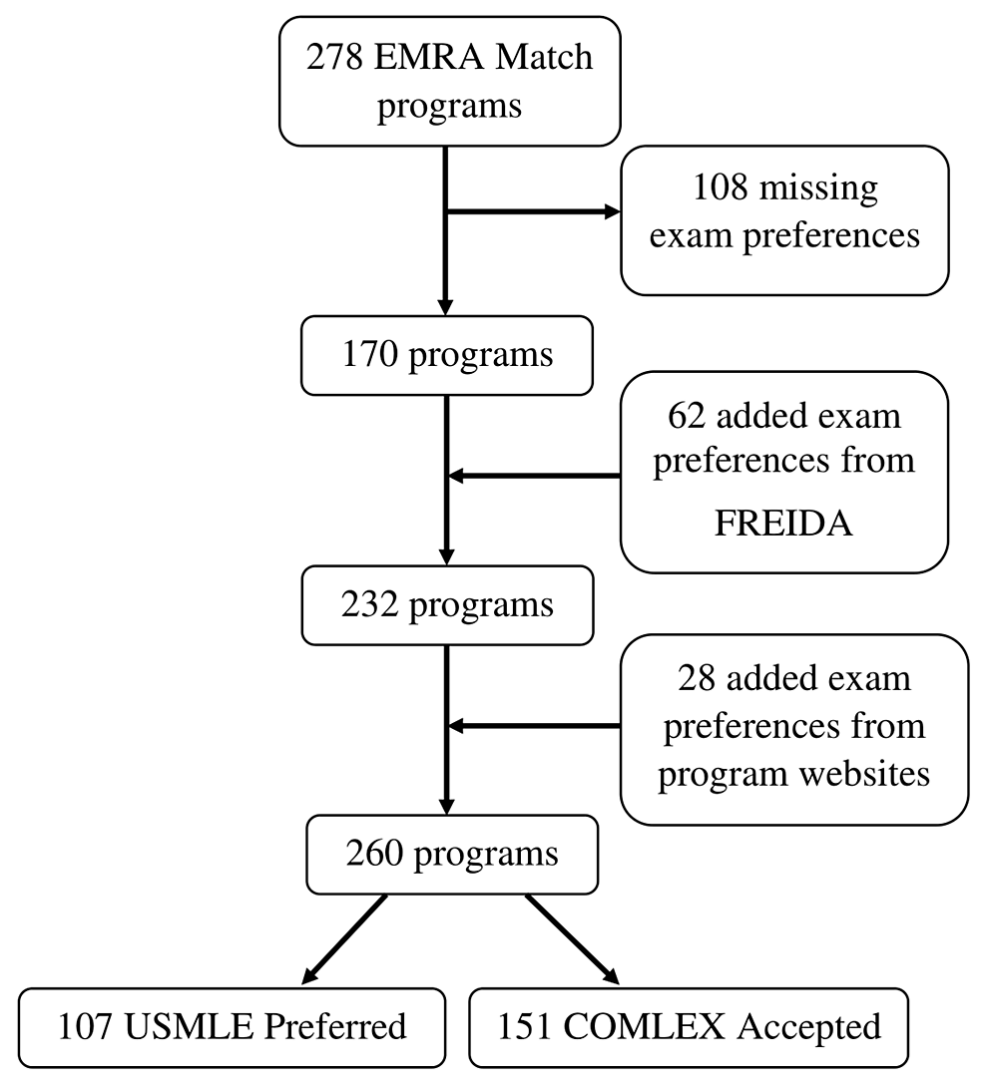
Study flow diagram illustrating program selection and exclusions. EMRA: Emergency Medicine Residents’ Association; USMLE: United States Medical Licensing Examination; COMLEX: Comprehensive Osteopathic Medical Licensing Examination; FREIDA: Fellowship and Residency Electronic Interactive Database Access

Of the 278 EM programs in the EMRA Match dataset, 108 had missing exam preferences. Exam preference was added for 62 programs from FREIDA and 28 programs from their individual websites, leaving 260 for analysis. One hundred fifty-one programs were classified as accepting COMLEX-USA alone, while 107 programs were classified as preferring the USMLE (Figure 1).

Program characteristics that differed between the groups included the educational environment of the primary training site (p <0.001), number of SLOE(s) needed for an interview (p = 0.042), ED shift length (p = 0.021), former AOA accreditation (p <0.001), percentage of osteopathic residents (p <0.001), annual ED volume (p = 0.001), number of intern positions (p <0.001), and number of elective weeks (p = 0.028) (Table 1).

**Table 1:** Emergency medicine residency program characteristics USMLE: United States Medical Licensing Examination; COMLEX: Comprehensive Osteopathic Medical Licensing Examination; SLOE: standardized letters of evaluation; AOA: American Osteopathic Association; ED: emergency department *p-value is <0.05.

	USMLE Preferred	COMLEX Accepted	p-value
n	107	151	
Region, n (%)			0.079
Midwest	21 (19.6)	43 (28.5)	
Northeast	26 (24.3)	49 (32.5)	
South	42 (39.3)	42 (27.8)	
West	17 (15.9)	17 (11.3)	
Missing	1 (0.9)	0 (0.0)	
Primary training site, n (%)			<0.001^*^
Community	29 (27.1)	81 (53.6)	
County	17 (15.9)	16 (10.6)	
University	57 (53.3)	41 (27.2)	
Missing	4 (3.7)	13 (8.6)	
Step 1 score minimum, n (%)			0.701
Declined to answer	16 (15.0)	20 (13.2)	
None	50 (46.7)	66 (43.7)	
Yes	34 (31.8)	49 (32.5)	
Missing	7 (6.5)	16 (10.6)	
Accepts step 1 failures, n (%)	30 (28.0)	48 (31.8)	0.611
Moonlighting allowed, n (%)	95 (88.8)	124 (82.1)	0.195
SLOE(s) needed for interview, n (%)			0.042^*^
None	2 (1.9)	12 (7.9)	
One	50 (46.7)	66 (43.7)	
Two	35 (32.7)	34 (22.5)	
Missing	20 (18.7)	39 (25.8)	
Program length, n (%)			0.328
3-year	81 (75.7)	123 (81.5)	
4-year	26 (24.3)	27 (17.9)	
Missing	0 (0.0)	1 (0.7)	
ED shift length, n (%)			0.021^*^
9 or less	45 (42.1)	43 (28.5)	
10	24 (22.4)	42 (27.8)	
12	9 (8.4)	27 (17.9)	
Variable	25 (23.4)	26 (17.2)	
Missing	4 (3.7)	13 (8.6)	
ED shifts per month, n (%)			0.135
15 or less	4 (3.7)	6 (4.0)	
16-18	42 (39.3)	73 (48.3)	
19-21	55 (51.4)	56 (37.1)	
22 or more	2 (1.9)	2 (1.3)	
Missing	4 (3.7)	14 (9.3)	
Former AOA accreditation, n (%)	4 (3.7)	46 (30.5)	<0.001^*^
Osteopathic residents, n (%)			<0.001^*^
0%	32 (29.9)	11 (7.3)	
1-10%	38 (35.5)	33 (21.9)	
11-20%	17 (15.9)	23 (15.2)	
21-60%	11 (10.3)	30 (19.9)	
61% or more	2 (1.9)	38 (25.2)	
Missing	7 (6.5)	16 (10.6)	
Annual ED volume			
Median (IQR)	93000 (71750-110000)	76000 (58260-100000)	0.001^*^
Missing, n (%)	4 (3.7)	12 (7.9)	
Percent of intern year off service			
Median (IQR)	42 (35.0-50.0)	42 (30.0-51.5)	0.598
Missing, n (%)	6 (5.6)	28 (18.5)	
Intern positions			
Median (IQR)	12 (10.0-14.5)	9 (6.0-12.0)	<0.001^*^
Missing, n (%)	0 (0.0)	2 (1.3)	
Elective weeks			
Median (IQR)	8 (6.0-12.0)	8 (4.0-10.0)	0.028^*^
Missing, n (%)	6 (5.6)	21 (13.9)	
Critical care weeks			
Median (IQR)	20 (16.0-22.0)	18 (16.0-22.0)	0.142
Missing, n (%)	4 (3.7)	17 (11.3)	

**Figure 2:**
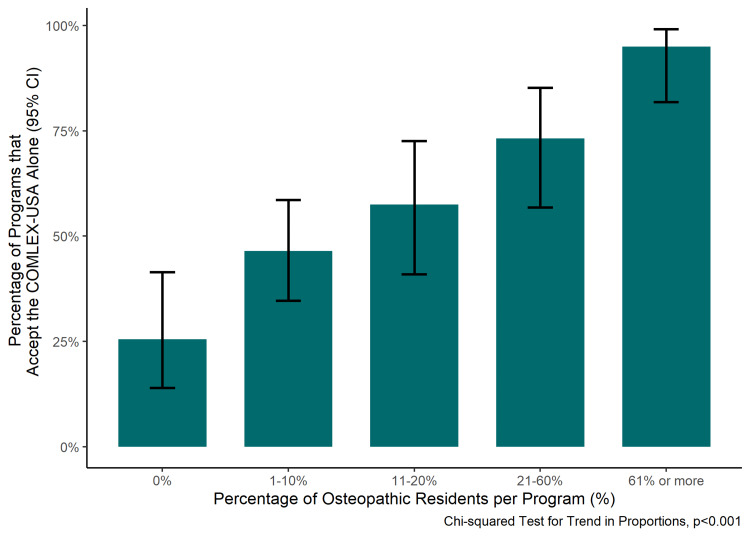
Proportion of emergency medicine programs that reported accepting the COMLEX-USA alone with 95% confidence intervals stratified by the proportion of osteopathic residents reported to be in the program. COMLEX-USA: Comprehensive Osteopathic Medical Licensing Examination; CI: confidence interval

The percentage of programs accepting COMLEX-USA alone increased as the percentage of osteopathic residents per program increased (p trend <0.001) (Figure 2).

## Discussion

After the Single Accreditation System transition, there are differences in program-level characteristics between EM residency programs that accept the COMLEX-USA alone for osteopathic applicants and those that prefer the USMLE. For example, community sites were more common among programs that accepted the COMLEX-USA alone compared to programs that preferred the USMLE for osteopathic applicants, 53.6% vs. 27.1%, respectively (Table 1). Also, the median annual ED volume was lower among programs that accepted the COMLEX-USA alone compared to those that preferred the USMLE, 76,000 vs. 93,000, respectively (Table 1). Therefore, osteopathic applicants who choose to forgo taking the USMLE should consider these differences when applying to EM residencies.

Prior work has shown that several variables we found to the contrast between programs with different exam preferences were important to EM applicants. In a survey of 261 EM-interested medical students, the most important variable in selecting a residency program was the educational environment of the primary training site (78.2% marked as most important) [19]. Other variables examined included the proportion of osteopathic and international medical graduates (42.1%), annual ED volume (56.7%), and program size (49.4%) [19].

Also, an increasing percentage of osteopathic residents per program was associated with an increasing percentage of programs accepting the COMLEX-USA alone for osteopathic applicants (Figure 2). Conceptually, it is likely that accepting the COMLEX-USA results in more osteopathic applicants to a particular program. However, having more osteopathic residents in a program may also attract more osteopathic applicants and provide more familiarity with the COMLEX-USA encouraging its acceptance.

Former AOA accreditation may also explain some of these differences. For instance, while the ACGME requires a minimum of 18 residents total, the AOA required only 16, which may explain the association between accepting COMLEX-USA and programs with fewer intern positions [20,21]. Although the minimum required annual ED volume of 30,000 for primary training sites was the same for both ACGME and AOA accredited EM residency programs, the smaller annual ED volume associated with accepting COMLEX-USA may be a byproduct of the lower minimum resident requirement from the former AOA programs [20,21].

Limitations

First, like all retrospective analyses, we can only assume correlation and not causation. Second, the information contained in EMRA Match, FREIDA, and program websites may be subject to reporting bias which may impact program-level responses. Last, we did not consider osteopathic recognition, an ACGME-accredited residency program status indicating dedication to the education of osteopathic principles and practice [22].

## Conclusions

EM residency programs that reported accepting the COMLEX-USA alone for osteopathic applicants differed from those that prefer the USMLE. Therefore, osteopathic applicants should consider these differences when deciding whether to take the USMLE.
